# A Unique Complication Associated with Concurrent Chemoradiation for the Treatment of Locally Advanced Head and Neck Cancer

**DOI:** 10.4137/cmo.s407

**Published:** 2008-04-01

**Authors:** Oren Cahlon, Ashok Shaha, Nancy Lee

**Affiliations:** 1 Department of Radiation Oncology, Memorial Sloan Kettering Cancer Center, New York, NY; 2 Department of Head and Neck Surgery, Memorial Sloan Kettering Cancer Center, New York, NY

**Keywords:** locally advanced head and neck cancer, complications, radiation, chemoradiation, toxicity

## Abstract

**Background:**

Concurrent chemoradiation is becoming an increasingly popular treatment for patients with locally advanced head and neck cancer. The full extent of treatment related complications has not been completely documented in the literature.

**Methods:**

We present the case of a patient treated with definitive intensity modulated radiation therapy and concurrent carboplatin and fluorouracil for a locally advanced oral cavity and base of tongue cancer.

**Results:**

The patient suffered acute grade 4 dermatitis and mucositis during treatment. One month after completion of treatment, the patient was found to have permanent adherence of the tongue to the buccal mucosa as a result of severe scar tissue formation.

**Conclusions:**

As more patients undergo chemoradiation for the treatment of locally advanced head and neck cancer, the full extent of treatment related complications are being identified. To our knowledge, this is the first report of chemoradiation for head and neck cancer resulting in adherence of the tongue to the buccal mucosa.

## Introduction

A major paradigm shift has occurred over the past two decades in the treatment of locally advanced head and neck cancer. Although many patients were cured using the traditional approach of surgery and postoperative radiation, functional outcomes were often suboptimal. This led investigators to pursue aggressive organ preservation protocols using combined chemotherapy and radiation.^1^ Chemoradiotherapy protocols have demonstrated equal survival rates compared to surgery while providing high rates of organ preservation and less treatment related morbidity.^2^–^4^ Nevertheless, the toxicity from this treatment can be debilitating with a myriad of early and late effects, resulting in a deleterious impact on patient quality of life.^5^–^7^

In this paper, we report on a 74 year old woman with a locally advanced, oral cavity and base of tongue (BOT) cancer treated with definitive intensity modulated radiation therapy (IMRT) and concurrent chemotherapy (5-fluorouracil and carboplatin). During treatment, she experienced grade 4 dermatitis and mucositis. Approximately one month after the completion of treatment, she developed adherence of the oral tongue to the buccal mucosa. To our knowledge, this particular toxicity has never been reported in the published literature.

## Case Report

A 74 year old female with no significant past medical history and no risk factors for head and neck cancer presented to the Memorial Sloan-Kettering Cancer Center in October 2005. She reported a several month history of difficulty placing her dentures, a painful lump towards the back of the tongue and a 20 pound unintentional weight loss. On examination, there was a large mass replacing the entire right side of the tongue extending from the tip of the tongue to the vallecula with infiltration of the right floor of mouth and submucosa. Biopsy of the mass identified squamous cell carcinoma. There were fixed right level II and level III lymph nodes.

**Figure 1:**
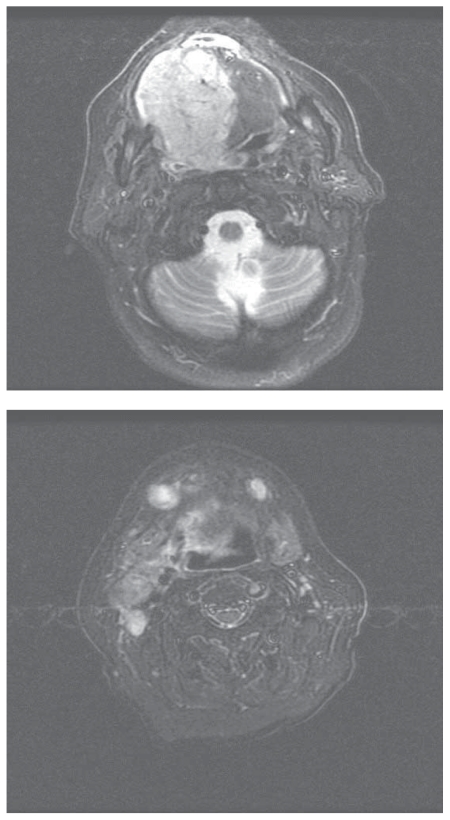
Pretreatment MRI. Axial fast spin-echo T2-weighted image with fat saturation at the level of the primary tumor and upper neck.

An MRI demonstrated a large, enhancing mass involving the right oral tongue and floor of mouth, crossing midline and extending posteriorly to the vallecula. There were bilateral enlarged cervical (Fig. 1). A PET/CT scan showed no evidence of distant metastases.

The tumor was staged as T4aN2cM0 oral cavity and BOT carcinoma; due to the extensive involvement of the oral cavity and BOT, it was difficult to determine the epicenter of the tumor. It was deemed to be resectable but would require a near-total glossectomy, base of tongue resection, pharyngectomy, laryngectomy and bilateral neck dissections. Furthermore, postoperative chemoradiation would likely be indicated for high-risk features. Given the anticipated morbidity of this treatment, definitive chemoradiation was recommended. A hearing evaluation identified high frequency hearing loss, precluding the use of cisplatin. Therefore, a decision was made to use three cycles of concurrent carboplatin (70 mg/m2) and 5-fluorouracil (600 mg/m2) administered every three weeks.

**Figure 2:**
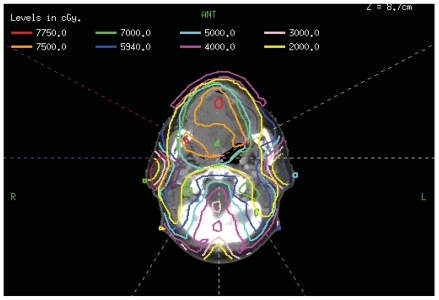
Isodose curves of an inverse IMRT plan displayed on the axial plane at the level of the oral cavity.

Radiation was delivered with a seven-field, dose painting, split field IMRT plan with primarily posterior beams (Fig. 2). The area of gross disease was prescribed 6996 cGy (2.12 cGy/fraction) and areas of high risk subclinical disease were prescribed 5940 cGy (180 cGy/fraction), all delivered in 33 fractions. The low neck received 5040 cGy in 28 fractions with a single anterior beam. The lips were not contoured as an avoidance structure. Isodose curves and dose volume histograms were generated and reviewed. Standard dose constraints were met for all critical structures. Parotid sparing was not possible due to the size of the mass. The mean dose to the oral cavity was unusually high (7444 cGy) due to the size and location of the tumor. The maximum PTV point dose was 7912 cGy (113% of prescription).

**Figure 3:**
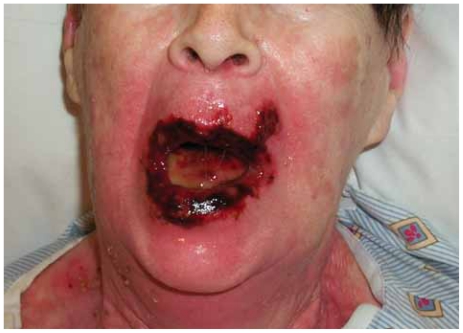
Phorograph at the start of week of six of treatment, just prior to receiving a treatment break.

A percutaneous gastrostomy tube (PEG) was placed prior to initiation of therapy for nutritional support during treatment. On 12/5/05 (day 1), the patient initiated radiation and chemotherapy. The second cycle of chemotherapy was administered as scheduled on day 22. During the fourth week of treatment, the patient developed grade 2 mucositis. She continued on treatment without major incident until the start of week 6 when she presented to clinic with severe full thickness ulcerations around the lips with spontaneous bleeding. In the oral cavity, there were confluent ulcerations with bleeding (Fig. 3) consistent with grade 4 dermatitis and mucositis. After a nine day treatment break, her symptoms subsided and she resumed radiation. She completed the remainder of radiation without difficulty on 2/1/06. The third cycle of chemotherapy was withheld. The patient had a slow decline in oral intake throughout treatment. By week six, she was entirely dependent on the PEG.

**Figure 4:**
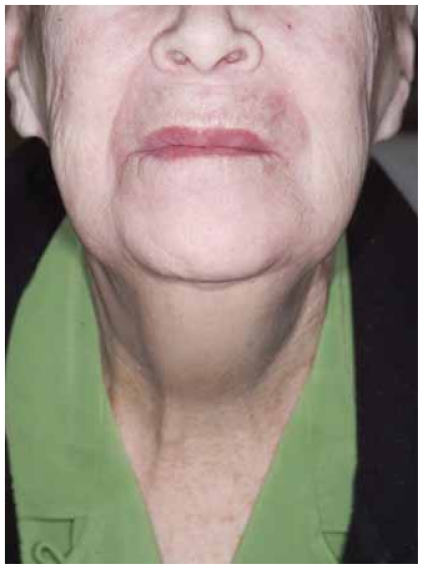
Photograph at the first followup visit five weeks after completion of treatment.

**Figure 5:**
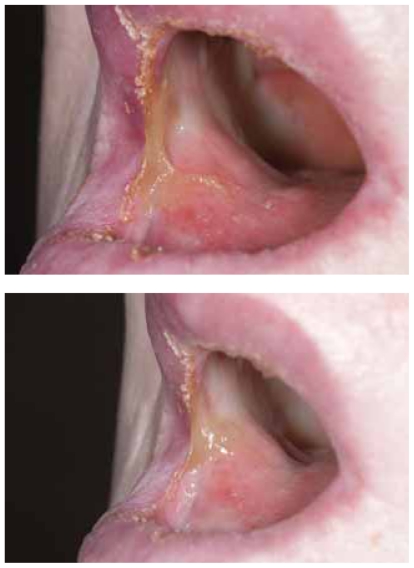
Photograph of the oral cavity at the first followup visit five weeks after completion of treatment. The right oral tongue is adhered to the right buccal mucosa.

She was seen for her first followup visit five weeks after completion of treatment. Her lips and perioral skin were completely healed (Fig. 4). She reported dysphagia and dysarthria. She was tolerating a liquid diet but was using the PEG for supplementation. On inspection of the oral cavity, the right side of the tongue was adhered to the right buccal mucosa (Fig. 5). Clinically, there was no evidence of disease. An MRI several weeks later showed no evidence of disease.

The consensus from the Head and Neck Tumor Board at MSKCC was to pursue a course of close observation. A lysis of adhesions was planned if there was no disease recurrence for at least six months. Unfortunately, within several months the patient developed widespread metastases. She died of progressive pulmonary disease in November. At the time of death, she was free loco-regional recurrence.

## Discussion

Aggressive chemoradiation regimens for the treatment of locally advanced head and neck cancer have been gaining popularity as a growing body of literature suggests equal survival between surgical and nonsurgical approaches.^1^–^4^ Intuitively, organ preservation should result in a better quality of life (QOL) by preserving breathing, swallowing and communicating functions. However, QOL in these patients is often compromised as a result of poor organ function. Several studies have shown that patients treated with organ preservation protocols often experience significant late toxicity resulting in severe physical, emotional and psychological effects and relatively low QOL scores.^6^–^8^ In a study from UCLA, researchers found equal QOL scores between patients who underwent radical surgery and postoperative radiation versus combined chemoradiation.^9^

As more patients receive and survive chemoradiation for head and neck cancers, a better appreciation of the long-term side effects associated with this treatment will be gained. In this paper, we report a unique complication in a patient treated with chemoradiation for a large of base of tongue carcinoma. During treatment, the patient experienced grade 4 mucositis and dermatitis. Such toxicity is known to occur in a small percentage of patients receiving concurrent chemoradiation. Shortly after the completion of treatment, however, she developed a complication that, to our knowledge, has not yet been described in the literature: adherence of the tongue to the buccal mucosa as a result of scar tissue.

Traditionally, radiation side effects have been categorized as either early or late and for many years the two were thought to be unrelated entities developing via different mechanisms. Recently, however, “consequential late effects” have been described.^10^–^14^ These were first observed with the introduction of aggressive treatment regimens using altered fractionation and combined modality protocols.^10^–^15^ In these situations, acute reactions fail to heal completely and persist into the “late” period.

We believe that the adherence of the tongue to the buccal mucosa described in the is report is analogous to pharyngoesophageal strictures, which are known to occur in up to 50% of patients receiving head and neck radiation.^6^ A study from the University of Miami showed that in patients receiving chemoradiation for head and neck cancer, those who experienced severe mucositis during treatment were more likely to develop esophageal strictures.^15^ Pharyngoesophageal strictures result from fibrosis, narrowing and muscle dysfunction. These strictures are usually detected several months after the completion of treatment. We hypothesize that the “stricture” described in this report resulted from a similar mechanism but was detected sooner than traditional strictures because of its anatomic location. The oral cavity is easily examined and adhesions in the oral cavity were immediately visible at the time of first followup. This is in contrast to esophageal strictures, which usually are not detected until a GI workup is pursued in patients with persistent dysphagia.

There is currently no consensus as to which chemotherapy regimen should be used in conjunction with definitive radiation as head to head comparisons have not yet been performed. Recent meta-analyses have shown that platinum-based chemotherapy is more effective than non-platinum containing regimens and that single agent therapy is equivalent to multi-agent therapy.^1^,^16^ Many institutions, including our own, use three cycles of cisplatin (100 mg/m^2^) administered every three weeks as the standard regimen. Trials with carboplatin and 5-FU have demonstrated comparable outcomes and toxicity compared to other regimens. However, 5-FU is known to cause mucositis and some consider this regimen to be more toxic. For non-platinum candidates, we consider Erbitux as an effective alternative at our institution. The obvious advantage to using concurrent Erbitux is that it does not increase the rate of mucositis compared to radiation alone and does not have a deleterious effect on QOL.^17^ It is possible that the use of 5-FU in this patient contributed to her complication. We now try to avoid using this regimen in patients who will receive a high oral cavity dose.

The use of IMRT as a technique to deliver high doses of radiation to target volumes and minimize the dose to nearby organs at risk has resulted in less long-term treatment related morbidity and enhanced quality of life for head and neck patients.^18^–^21^ One potential pitfall of IMRT, however, is that it often results in dose inhomogeneity and can lead to areas within the target or immediately outside of it receiving more than the prescription dose. In order to minimize this, it is critical to carefully evaluate dose volume histograms and volumetric dose distributions which will identify any “hot spots” in the PTV or immediately outside of it. In this particular report, the maximum point dose was 113% of prescription and there were two small areas in the oral cavity which received 111% (7750 cGy) (Fig. 2). These areas, however, did not correlate anatomically with the site of fibrosis on the right buccal mucosa. Therefore, we feel it is unlikely that the inhomogeneity of the plan contributed to the toxicity. Nevertheless, the value of using IMRT in this case, when normal tissue sparing was not attempted, is debatable. Using parallel opposed beams may have resulted in a more homogeneous dose distribution. In addition, due to the use of multiple beams with IMRT, it is not unusual to have “dose dumping” outside of the target area. This results in a higher volume of surrounding tissue receiving low dose radiation than with conventional radiation. For example, the lips in this case received a higher dose than would be expected with a conventional plan where the lips would have been completely out of the field. Therefore, IMRT should only be used for cases in which normal tissue sparing is the feasible. As a result of this event, we now often contour the lips as an avoidance structure when using IMRT to treat oral cavity and large oropharynx tumors.

Our preference has been to use dose-painting in conjunction with IMRT. With this technique, the area of gross disease receives a larger dose per fraction (2.12 Gy) and areas of subclinical disease receive lower doses (≤1.8 Gy). We have used this fractionation to treat multiple head and neck sites at our institution for several years with excellent tumor control and without unexpected toxicity.^22^–^23^ Altered fractionation results in higher rates of toxicity than standard fractionation. When altered fractionation is used with concurrent chemotherapy, toxicity is quite severe. Perhaps dose painting IMRT (which is a form of altered fractionation) with concurrent chemotherapy also increases toxicity. More time will be needed to determine the full extent of late effects with this regimen. It is unlikely that fraction size contributed to this particular toxicity.

We have not routinely used amifostine in patients being treated with concurrent IMRT and chemotherapy. Although amifostine has been proven to reduce the rate of acute and late xerostomia,^24^ its ability to decrease mucositis is conflicting.^25^, ^26^ Perhaps in cases such as this with large target volumes, a radiation protector such as amifostine should be considered.

In conclusion, this report contributes to the growing body of literature describing the acute and late effects associated with head and neck radiotherapy. As more patients are treated with aggressive organ preservation protocols, it is important to characterize the morbidity of treatment and to study its impact on quality of life. We must not forget that the goal of such regimens is organ function, not just organ preservation. Careful attention should be given to radiation technique, dose, fractionation and concurrent systemic therapy to minimize potential toxicity.
